# Artificial Intelligence in IR Thermal Imaging and Sensing for Medical Applications

**DOI:** 10.3390/s25030891

**Published:** 2025-02-01

**Authors:** Antoni Z. Nowakowski, Mariusz Kaczmarek

**Affiliations:** Biomedical Engineering Department, Gdansk University of Technology, Narutowicza 11/12, 80-233 Gdańsk, Poland; markaczm@pg.edu.pl

**Keywords:** medical thermography, IR thermal imaging, machine learning, artificial intelligence methods

## Abstract

The state of the art in IR thermal imaging methods for applications in medical diagnostics is discussed. A review of advances in IR thermal imaging technology in the years 1960–2024 is presented. Recently used artificial intelligence (AI) methods in the analysis of thermal images are the main interest. IR thermography is discussed in view of novel applications of machine learning methods for improved diagnostic analysis and medical treatment. The AI approach aims to improve image quality by denoising thermal images, using applications of AI super-resolution algorithms, removing artifacts, object detection, face and characteristic features localization, complex matching of diagnostic symptoms, etc.

## 1. Introduction—Temperature and Heat Flows in Medical Applications

Temperature—T—is one of seven basic metrology parameters defined in the International System of Units (S.I.) as thermodynamic temperature with the unit Kelvin (K). In most of the world, the Celsius scale (°C) is used for common temperature measurements in daily life. It is an empirical scale that developed historically, which led to its zero point, 0 °C, being defined as the freezing point of water, and 100 °C as the boiling point of water, both at atmospheric pressure at sea level. In medicine, temperature is one of the basic diagnostic parameters that allow for a quantitative description of a patient’s state and a measure of the conditions of life in the environment. Most important for diagnostics are thermal gradients—the temperature difference unit is 1 K = 1 °C—and time constants of transient thermal processes in seconds.

The aim of this paper is to present fascinating developments of technologies devoted to improved thermal diagnostics in medicine. This started in ancient times when Hippocrates discovered that wet mud used on the skin allows one to observe fast drying over a tumorous swelling. This was the first method of thermal imaging for medical diagnostics! Today, everyone knows that high temperatures are important for diagnosing illness; mothers diagnose children’s fever just by holding a hand on the forehead. It is worth underlining that temperature is a functional physiological parameter, though structural information is required to recognize the reason for illness properly. The importance of temperature measurements in medicine was broadly discussed in the first issue of the journal *Lancet* in 1916 [1].

Normal body temperature due to thermoregulation processes (homeostasis) varies by person, age, activity, and time of day in a relatively narrow range from 36.1 °C to 37.2 °C. Any dysfunction of the living organism may be manifested by hypo- or hyperthermia, causing characteristic temperature distribution over the body’s skin, allowing differentiation of physiological processes inside a patient. Therefore, registration of temperature distribution over the skin allows for important patient diagnostics.

There are practical limitations for temperature measurements in a patient due to physiological processes: hyperthermia exceeding 42 °C leads to tissue necrosis, and low temperatures are dangerous for life, leading to hypothermia below ~30 °C. Therefore, body temperature measurements within this range are of the highest diagnostic value and determine the required technical properties of diagnostic instrumentation, while control of the ambient environment requires measurements in the range of −50 to +50 °C or even higher temperatures. The accuracy of measurements and thermal resolution in medical thermometers should be at least 0.1 °C.

Static body temperature (ST)—at thermodynamic equilibrium—used to be measured by single sensors, such as glass thermometers and electric and electronic sensors. The latter include thermoresistors, thermopiles, thermistors, semiconductor thermometers, and recently, infrared hand-held thermometers—radiometers. The quantitative approach to describe the state of a patient by temperature started with the invention of the clinical-maximum glass thermometer, which was introduced to medical practice in 1865–1870 by Karl Wunderlich [1].

Essential progress in thermal imaging was noted in the 1960s by the adaptation of military infrared thermal cameras to medical diagnostic applications; see, e.g., [2]. The state of the art in thermal imaging in medicine is summarized in [3]. Unfortunately, limited knowledge of measurement conditions in early IR thermal imaging and complicated heat flows in humans caused issues such that after the enthusiastic welcome of this method, the medical staff was rather disappointed due to many false interventions in breast cancer treatment. Recent advances in machine learning and a rise in confidence in using extended databases again promote a high interest in this technology.

Recently, scientific and technical progress has concentrated on the analysis of transient heat flows and thermal imaging in multimodality diagnostic applications, possibly due to advanced mathematical tools and algorithms introduced to 2D, 3D, and 4D models and supported by AI methods. The role of three professional organizations in the promotion of new technologies is underlined here:

(a) IEEE EMBS https://www.embs.org (accessed on 27 December 2024)—Engineering in Medicine and Biology Society, the largest organization of engineers in medicine, founded in 1952 and publishes several journals, including open access *Engineering in Medicine and Biology*;
(b) QIRT http://qirt.gel.ulaval.ca (accessed on 27 December 2024)—Quantitative Infrared Thermography Organization, founded in 1992 and publishes the *QIRT Journal*;
(c) EAT http://www.eurothermology.org (accessed on 27 December 2024)—European Association of Thermology, medical international society publishing *Thermology International* (open access), founded in 1990.

The motto of the QIRT Organization is cited as follows:

“Researchers have been familiar with infrared thermography for many years now. This technique took many fields by storm, sometimes to wither shortly after. While the beauty of thermographic images and the ease of getting them could explain the success of this technique, the difficulty of getting accurate measurements using “thermal cameras” has caused a certain disaffection toward it”.

In the following sections, advances in IR thermal imaging technology are summarized, problems of model-based methods are briefly described, and machine learning methods using standardized procedures and big databases are discussed. This review is summarized by indications and enumeration of advised solutions in clinical practice as well as addressing problems still to be solved.

The literature on this subject is very broad as every year, more than 1000 original publications and other positions are published; see, e.g., [4,5]. The knowledge in the field is summarized in several monographs; therefore, here, we recommend a few ones: on the basics of IR thermal imaging methods in medicine [6,7,8]; on heat transfer and temperature diagnostics in medical applications [9,10,11]; and on thermal image data treatment, including AI methods [12,13].

## 2. Advances in IR Thermal Imaging Methods in Medicine

Based on the cited monographs [3,6,7,8,9,10,11], advances in IR thermal imaging in medicine within the period 1960–2020 are summarized here. It should be underlined that any progress in this technology is driven by the military industry on one side and academic research in technology and medicine on the other side. Novel artificial intelligence methods have been present in IR thermal imaging roughly since 2020!

In 1947, the US military developed the first infrared line scanner (one image took an hour to produce). In 1960, a military thermal camera (1 thermal image/minute) was adapted for medical research. In 1963, the first medical thermography conference was held in New York, where one of the authors concluded the following: "All that has been revealed by this technique is nothing compared with what is left to be discovered." This statement seems to still be valid today! In 1966, the first real-time commercial thermal imager appeared, followed by an explosion of new constructions based on several discoveries in thermal and semiconductor detector technologies.

There are two infrared detector technologies applied in thermal imaging:

- Thermal detection, non-selective;
- Photon detection, based on the direct excitation of photons into, e.g., electrons, with a long wavelength limit of sensitivity.

Thermal detection uses the relation between conductivity, capacitance, thermoelectric and pyroelectric phenomena, etc., depending on detector temperature. Thermal detectors are not selective, cover all electromagnetic spectrum, and do not require cryogenic temperatures for operation. Such detectors are of limited speed—the time constants of detectors are limited by heat flows to milliseconds and are slow compared to photon detection, lasting microseconds or less.

Photon detection may be manifested by the electric charge accumulated, the current flow, voltage, or the change in conductivity that is proportional to irradiation. This category contains many semiconductor detectors, such as InSb, HgCdTe, quantum well detectors (QWIPs), and many others. All require cooling to cryogenic temperatures, e.g., 77 K of liquid nitrogen. Newer photon infrared cameras are operated at elevated temperatures using more handy solid-state thermoelectric coolers (Peltier cooler) and sterling coolers.

The IR spectrum of thermographic cameras used for medical applications covers the following ranges: Mid-Wave Infrared (MWIR), 3 μm to 5.5 μm, based on mainly InSb detectors, and Long-Wave Infrared (LWIR), 8 μm to 12 μm, using mainly HgCdTe and QWIP detectors and thermal FPA. The use of Very-Long-Wave Infrared (VLWIR), 12 μm to 25 μm, may be noticed in very expensive cameras applied for research.

During the last 60 years, several models of thermal cameras applied to medical diagnostics have been classified according to the number of elements contained:

∘ Single-element detectors, or detectors with only a few elements; a two-dimensional advanced mechanical scanner using mirrors or other solutions, e.g., prisms, was applied to generate a two-dimensional image.
∘ Array detectors (FPA—focal plane array) that do not require any scanning mechanism for acquiring the two-dimensional picture. Nowadays, only this generation of cameras is in use for medical applications.

Thermal cameras based on cryogenically cooled or thermoelectric-cooled detectors are characterized by high electrical power consumption resulting in short battery life; relatively long cooling-down time (usually more than a few minutes); limited cooler lifetime, usually a few thousand hours; larger size and weight; high cost—USD 30 k–>USD 100 k.

Systems based on the InSb cryogenically cooled detectors operating in the 3 μm to 5.5 μm spectral band have higher BLIP background-limited performance (low NEDT (noise equivalent temperature difference) values) compared to LWIR imagers, providing better thermal and spatial resolutions.

In the 1990s, major progress in detector technology—the introduction of high-resolution, cooled, and uncooled focal plane arrays (FPAs)—allowed for dramatic improvement in thermal imaging in terms of speed, resolution, and cost of IR thermal cameras.

Uncooled microbolometer detectors collect the radiation, usually in the 8 μm to 12 μm spectral band, limited by optics. This spectral band provides better penetration through smoke, dust, water vapor, etc., because the wavelength is much longer than the 3 μm to 5 μm spectral band. Cameras are handy, small, and lightweight; nowadays, miniature uncooled microbolometer thermal cameras may be attached directly to smartphones, provide real video output immediately after power is on, require very low power consumption relative to cooled detector thermal imagers, and allow for a long time of life.

During the last 30 years, remarkable progress has been observed in electronics and microprocessors, allowing for high sensitivity and higher resolution of images. Systems with improved signal-to-noise and image acquisition speed are available for reasonable prices. Today, images are digitized, stored, manipulated, and processed on board the camera. Removable PCMCIA cards can store more than a few thousand radiometric infrared images for recall, analysis, or archiving. Intuitive Windows-based software for reporting, archiving, and analyzing thermal images is in use in practically any IR thermal camera and is loaded on laptop computers and pocket PCs.

It is expected that the technology will continue to develop, particularly in the area of improved detector performance and reduced noise equivalent temperature difference. Recently, multimodality RGB and IR thermal uncooled arrays have been combined in one camera, and even multispectral solutions have appeared on the market. Inexpensive miniature cameras as well as high-performance professional cameras with arrays of 640 × 512 and even 1920 × 1536 pixels are already available. Cameras allow the integration of multispectral infrared, visual and blended Images, and live infrared images to be overlaid directly onto the visual camera pictures and improve the storage of images, sound, and data. Still, the accuracy of temperature measurements using IR thermal cameras is limited to 1–2 °C. Therefore, it seems more important to use differential data to allow a comparison of images collected in symmetric body parts or in flowing time. The parameter NEDT, even in inexpensive cameras, may be <50 mK and <15 mK in cooled FPA cameras.

Great interest in modern artificial intelligence tools gives hope that the future of IR thermal diagnostics will develop with the help of modern mathematic tools using personal computers and knowledge hidden in extended databases, which is discussed in Section 4. Advances in IR thermal imaging are summarized in Table 1.

## 3. Problems of Thermal Image Acquisition and Reconstruction of Diagnostic Parameters

Early thermographic imaging techniques were static (ST), providing only a snapshot of temperature distribution on the body’s surface under stable thermodynamic conditions. Establishing and agreeing upon the conditions for static thermographic imaging in medical diagnostics took several decades, resulting in the development of the “golden standard” for ST [14,15]. The standard includes the following key measures:

- Preparation of the Patient:
  ∘ Patients should avoid stimulants and medications prior to the test.
  ∘ Physical activities that could stimulate the body should be avoided.
  ∘ The skin in the area of interest must be free of ointments, creams, or other substances.
  ∘ Patients should remain in the waiting or examination room for at least 15 min before testing to allow stabilization of thermal conditions.
  ∘ The areas of interest should be exposed during this period to achieve thermodynamic equilibrium.
- Technical Specifications of Diagnostic Equipment:
  ∘ Use an appropriate infrared camera with specified geometric and thermal resolution (at least 0.1 K).
  ∘ Camera positioning: perpendicular to the field of interest, at a minimum distance of 0.5 m.
  ∘ Scan frequency: the rate at which images are recorded over a given period.
- Climatic Conditions in the Testing Room:
  ∘ The room temperature should be maintained between 18 and 23 °C (maximum 25 °C), stabilized with an accuracy of at least 1 °C.
  ∘ No ventilation, radiation sources, or heaters should influence the temperature of the patient’s body.
  ∘ Controlled and constant humidity in the test room is essential.
- Databases and Image Cataloging:
  ∘ Comprehensive databases and catalogs have been developed to standardize the conditions for storing and processing medical images of specific regions of interest (ROIs) on the patient’s body [16].
  ∘ However, the DICOM (Digital Imaging and Communications in Medicine) standard for thermal imaging has not yet been implemented.

In the end–beginning of the XX/XXI centuries, transient thermal processes were introduced to medical diagnostics, based on forced thermal flows and reconstruction of thermal model parameters [17,18,19,20,21], called active thermography and Active Dynamic Thermography (ADT) or Thermographic Signal Reconstruction (TSR). This was an important step towards quantitative thermal diagnostics of highly improved resolution. So far, no agreed-upon guidelines have been defined in the diagnosis of a patient after using external thermal stimulation and analysis of resulting thermal transient processes. Thus, people using dynamic thermography methods practically apply their own diagnostic instrumentation and algorithms, obtaining “some” results, but there are no agreed-upon procedures allowing for reliable assessment of the applied approach. The proposal to solve this problem was discussed in [22] and is repeated here. The state of the art and conclusions are presented below.

### 3.1. Theory

Thermal diagnostics relies on analyzing thermal transient processes using thermal models correlated with physiological processes. In theory, this involves identifying the parameters of an equivalent thermal model for the tested object, specifically within the defined region of interest (ROI). This task is particularly challenging in medical diagnostics due to several factors: first, the structures under examination are often complex and poorly defined; second, measurement and excitation access to the ROI are typically unilateral, limiting data acquisition; and third, the initial and boundary conditions required for reconstructing equivalent thermal model parameters are usually unknown and obscured by biofeedback mechanisms that introduce variability. This type of issue is called “direct problem” solving. The “direct problem” in thermal diagnostics involves determining the temperature distribution over time and space based on assumed boundary conditions and known model parameters. This is also feasible when external thermal excitation is applied.

In biological applications, solving the direct problem requires addressing heat flow in three-dimensional space. The analysis often utilizes the Pennes “biologic heat flow equation” [23], which remains a foundational model for heat transfer in biological tissues.

(1) $$c_{t}\cdot \rho_{t}\frac{\partial T\left(x,y,z,t\right)}{\partial t}=k\cdot \nabla^{2}\cdot T\left(x,y,z,t\right)+Q_{b}+Q_{m}+Q_{e}$$

where T(x,y,z,t) is temperature distribution [K] at the moment t [s]; (x,y,z)—coordinates in 3D space; k—thermal conductivity [Wm^−1^K^−1^]; c_t_—specific heat [Jkg^−1^K^−1^]; ρ_t_—material (tissue) density [kg m^−3^]; Q_b_ [Wm^−3^]—heat power density delivered or dissipated by blood; Q_m_—heat power density delivered by metabolism; Q_e_—heat power density delivered by external sources.

**Figure 1:**
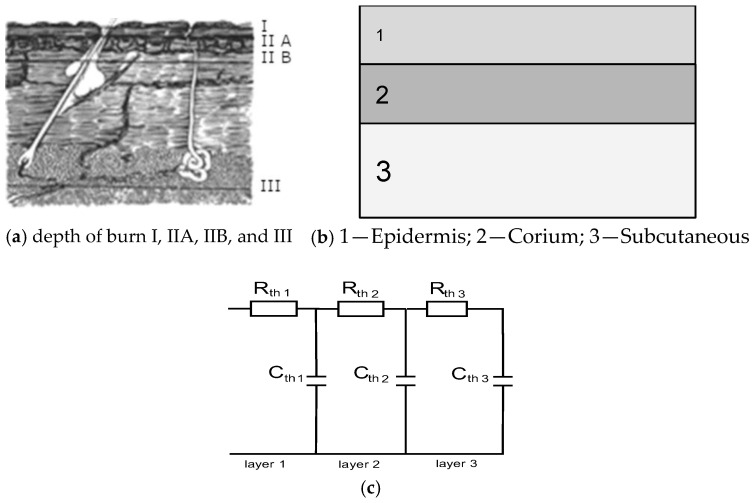
(**a**) Anatomy of the skin (from [10])—depth of burn; (**b**) three-layer structural model; (**c**) equivalent thermoelectric model [22].

To determine the volumetric temperature distribution, three primary approaches are commonly employed, as illustrated in Figure 1:

- Analytical Methods: Analytical solutions are applicable only to very simple structures with well-defined shapes. Due to their limitations in handling complex geometries, these methods are primarily used to provide rough estimates.
- Numerical Methods: Numerical approaches, often based on the Finite Element Method (FEM), are widely used for solving heat transfer problems. Several commercial software packages integrate FEM for heat flow analysis, offering features like model mesh generation and specialized thermal problem modules. Examples include general-purpose mathematical software such as ANSYS, COMSOL, MATLAB, and Mathematica.
- Equivalent Thermoelectric Models: This approach is the simplest for practical applications. It uses equivalent electro-thermal parameters to model thermal behavior, such as the example shown in Figure 1c, illustrating an electro-thermal model applied to skin burns [22].

FEM methods allow the solution of 3D heat flow problems in time. The functional variability of thermal properties and nonlinear parametric description of tested objects may be easily taken into consideration. Unfortunately, there are basic limitations concerning the dimension of a problem to be solved (complexity and a high number of model elements), which should be taken into account from the point of view of the computational costs.

**Figure 2:**
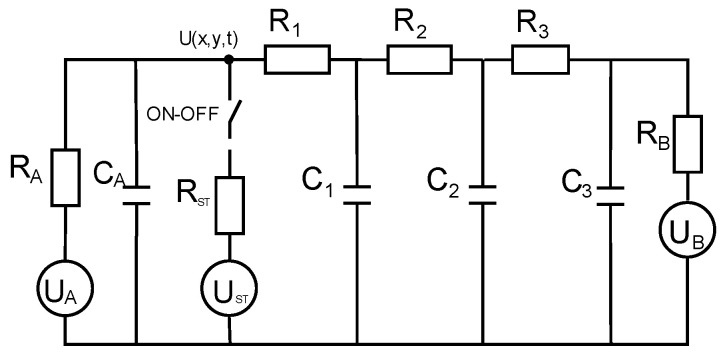
Electric equivalent circuit simulating thermal processes at the ROI surface, where U(x,y,t) is the voltage of a temperature value at a pixel x, y at time t; U—voltage sources representing ambient A, stimulation ST, and body B temperature (boundary conditions); Ri—thermal resistances; Ci—thermal capacitances [22].

In medical diagnostics, the practical question is solving the reverse problem—reconstructing model parameters. Several conditions are necessary for obtaining reliable results, which are discussed here for a model of the skin in thermal transients, as in Figure 2.

In static thermography under equilibrium conditions, the surface temperature distribution T(x,y,0) = U(x,y,0) depends on the following factors: boundary ambient temperature (U_A_)—typically around 20 °C; internal body temperature (U_B_)—typically around 37 °C; tissue property distribution—represented by nonlinear thermal resistances; heat exchange with the environment (R_A_)—combines radiation heat exchange and air conduction. Thermal capacitances come into play only in transient heat flows. Notably, static temperature plays a crucial role as an initial boundary condition in thermal analysis.

When a stimulation source (U_ST_) is applied, it enables the recording of thermal transients, represented by a series of thermograms over time (U(x,y,t)). These recordings allow for the reconstruction of thermal capacitances (C_i_) and thermal resistance components (R_i_).

It is important to emphasize that the activation and deactivation of the stimulation source alter the structure of the thermal circuit, leading to different heat flow patterns. Additionally, heating and cooling modify boundary conditions and can even change structural parameters (e.g., dimensions of the thermal model) because the depth of heat penetration depends on the duration of the excitation; longer excitation times result in deeper thermal effects.

The most commonly reconstructed parameters are those associated with the equivalent exponential Equations (2) and (3). These parameters include the following: steady state temperature component Ts(x,y) = T(x,y,0); delta coefficients (Δ), representing the increases or decreases in temperature T over time; thermal time constants, characterizing the temporal response of the tissue to thermal changes. These parameters allow the quantitative description of the examined tissue using time constants and exponential terms, providing valuable insights into its thermal and physiological properties. We assume the following equations:

(2) $$T\left(x,y,t\right)=T_{S}\left(x,y\right)+\sum_{j=1}^{m}∆T_{j}\left(x,y\right)\cdot \left(-e^{-\frac{t}{\tau_{j\left(x,y\right)}}}\right)$$

for the natural heating phase after switching off the cooling stimulus. The natural cooling phase after switching off the heating stimulation source is described by a very similar equation, Equation (3)—note the lack of the minus sign in the last component:

(3) $$T\left(x,y,t\right)=T_{S}\left(x,y\right)+\sum_{j=1}^{m}∆T_{j}\left(x,y\right)\cdot \left(e^{-\frac{t}{\tau_{j\left(x,y\right)}}}\right)$$

where T(x,y,t)—temperature of the pixel (x,y) for the time t; T_s_(x,y)—static temperature of the pixel (x,y); ΔT_j_(x, y)—values of temperature changes caused by the excitation stimulus; τ _j_(x,y)—time constants. Formally, both equations are described by the same symbols, but practically, the values of the parameters are different as the heat flows are different for cooling and for heating, for both the excitation phase and for the recovery to equilibrium phase!

Temperature transients in the region of interest (ROI) are registered with limited accuracy, and the reconstruction of structural parameters based on thermographic image sequences is constrained. To achieve sufficient compliance between the model and the measured data, it is common to determine only two exponential components (m = 2). The measurement data obtained from the thermographic camera are fitted to exponential models using algorithms such as the simplex method, Levenberg–Marquardt, or other optimization techniques that minimize error functions. Preprocessing techniques such as low-pass filtering or median filtering can be applied to the data to improve the quality of the fit to the replacement model. These methods help reduce noise and improve parameter estimation accuracy, ensuring better alignment with the theoretical model.

Similarly, transient changes on the tissue surface can be characterized by the TSR (Thermographic Signal Reconstruction) method. The method assumes that the temperature changes for the area without defects should be consistent with the one-dimensional Fourier heat flow equation and that the area above the defect will have deviations from this course. One can write the Fourier equation in the logarithmic domain [24] as

(4) $$ln\left(\Delta T\left(x,y,t\right)\right)=ln\left(\frac{Q}{e}\right)-\frac{1}{2}ln\left(\pi \cdot t\right),$$

where Q is heat power density and e is effusivity. For the data space transformed in this way, one may adjust the data to the n-degree polynomial:

(5) $$ln\left(\Delta T\left(x,y,t\right)\right)=a_{0\;}+a_{1\;}ln\left(T\left(x,y,t\right)\right)+a_{2\;}[{ln\left(T\left(x,y,t\right)\right)]}^{2\;}+...+a_{n\;}[{ln\left(T\left(x,y,t\right)\right)]}^{n\;}$$

where a_i_—coefficients of the polynomial; n—order of the polynomial; T—temperature; t—time.

Typically, the order of the polynomial is 4–5. As the last step of the procedure, the first and second derivatives of the parameter changes after time are determined.

One practical problem should be underlined in pulse thermography—the choice of excitation time should be carefully analyzed! The simplest model of heat penetration shows that so-called heat diffusion length L is a function of material properties (alfa (α)—tissue diffusivity) and time t,

(6) $$L={2\left(\alpha \cdot t\right)}^{1/2}$$

which means that the excitation time should be chosen according to expected model depths; on the other hand, one should assume that the equivalent thermal model may be changed with time excitation!

As in the electric circuit theory, there are possible analyses in time and frequency domains, with all the specific features of those de facto equivalent methods. However, in equivalent thermal models used in medical thermography, the situation is more complicated due to thermoregulation processes and safety conditions limiting the excitation energy to values safe for a patient.

Thermal Tomography—TT—requires the most complicated procedure of model parameter modification in the process of the comparison of model simulation results and measurement data [21].

### 3.2. Practice

**Figure 3:**
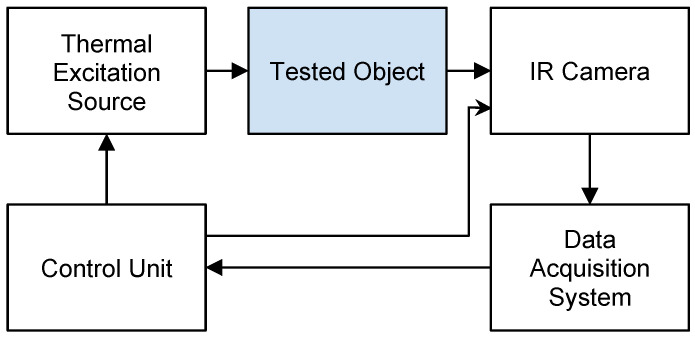
Block diagram of basic ADT/TSR/TT measurement procedure [22].

The general measurement concept for Active Dynamic Thermography (ADT), Thermal Tomography (TT), and Thermographic Signal Reconstruction (TSR) or Phase Pulse Thermography (with a sinusoidal type of excitation) is illustrated in Figure 3. The process involves the following steps: In initial temperature registration, the steady-state temperature distribution of the tested surface (ROI) is recorded using an infrared (IR) camera. Next, a controlled thermal excitation (heating or cooling) is applied to the ROI. Temperature transients at the ROI surface are recorded, particularly during the recovery phase after the excitation is switched off. An external thermal excitation source operates under fully controlled conditions. A control unit synchronizes the excitation process with temperature registration to ensure precise data capture. Data collection and analysis are the final steps. For pulsed excitation, diagnostic measurements focus on the recovery phase to the initial conditions. All temperature data are stored in the data acquisition system for subsequent computer analysis and determination of model parameters. This process ensures accurate and reproducible thermal measurements for diagnostic purposes.

In medical applications, working with living patients introduces challenges such as unintentional movements (e.g., breathing) that must be addressed. This requires image synchronization and correction, ensuring that sequential images are aligned over time. ROI consistency involves maintaining the region of interest (ROI) in the same position across all images in a series, even when data are collected on different days (e.g., during wound healing studies); do not forget about camera movement adjustments and correcting for any shifts or motions of the camera during imaging.

#### Thermal Tomography Procedures

**Figure 4:**
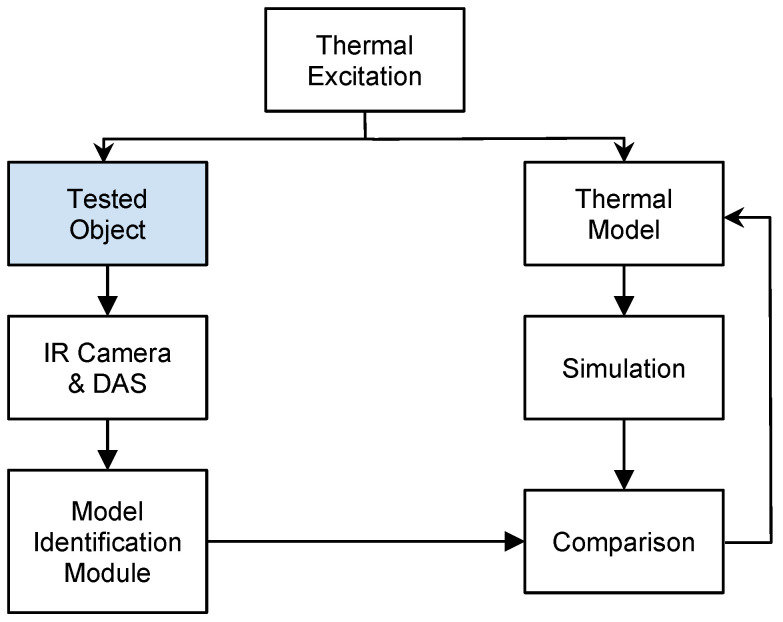
Thermal Tomography procedure for reconstruction of a tested structure’s properties [22].

Thermal Tomography presents additional complexity, as it involves reconstructing the dimensions and material properties of 3D structural thermal models. The typical procedure for reconstructing model parameters is shown in Figure 4. It includes the following steps: In thermal excitation, a controlled thermal excitation (physical or simulated) is applied to the tested structure and its corresponding thermal model simultaneously. Next, temperature transients on the object’s surface are recorded using an infrared (IR) camera. In parallel, the same thermal excitation process is simulated for the thermal model. For each pixel, the recorded temperature time course is identified and compared with the simulated transient. If discrepancies exist between the measured and simulated results, the thermal model is modified iteratively. Adjustments continue until the simulation results converge with the experimental data. The final step is model validation. Once the simulation and measurement results are convergent, the reconstruction procedure is complete. At this stage, the model is considered to represent the tested structure with a high degree of confidence. This iterative and precise process ensures that the reconstructed thermal model accurately reflects the physical properties and behavior of the tested structure.

Unfortunately, this reconstruction procedure is rather long and not always successful as the problem is mathematically ill posed, boundary conditions are not always properly identified, and the accuracy of measurements is limited. Until now, TT has been regarded as a research tool, but it has not been applied in clinics.

**Figure 5:**
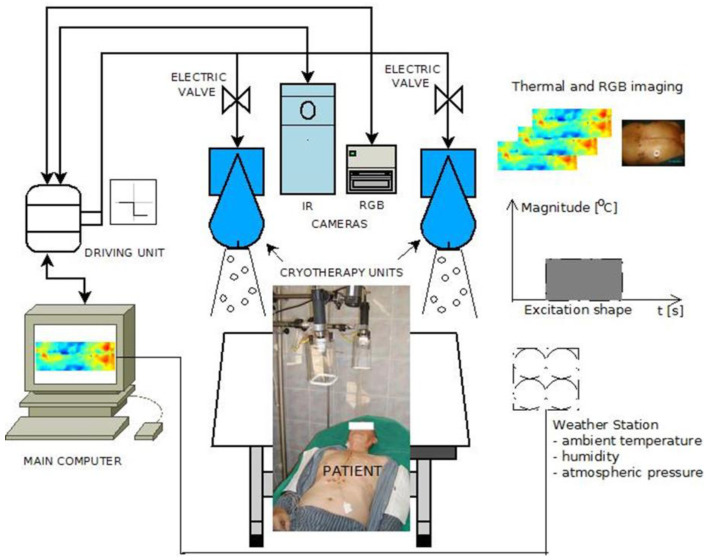
Block diagram of the stand for research with the ADT, TSR, and TT methods [22].

As a practical example of the possibilities of using the Active Dynamic Thermography technique in medical diagnostics, a prototype measurement system developed at the Department of Biomedical Engineering of the Gdansk University of Technology is presented in Figure 5. The system comprises the following components:

- Main Computer: Controls the entire operation, managing excitation sequences and acquiring data and reconstruction of thermal replacement model parameters.
- Driving Unit (Controller): Adjusts control signals, ensuring appropriate voltage levels and I/O current efficiency for operating excitation devices (e.g., cryotherapy equipment).
- Symmetrical Thermal Excitation System: Provides controlled thermal stimulation (in our case the cryotherapy CO_2_ units).
- Thermographic Camera: Captures thermal images of the region of interest (ROI).
- RGB Camera: Records visible-spectrum images for alignment and analysis.
- Additional equipment, such as a weather station for monitoring environmental conditions—temperature and air humidity.

The main computer manages the sequence of excitation signals and synchronizes the acquisition of RGB image data and thermographic sequences. The controller ensures precise signal adjustment and the operational efficiency of excitation devices.

Diagnostic instruments are equipped with specialized software packages that facilitate easy manipulation of settings and at least semi-automatic registration of images and data. These tools streamline the acquisition, processing, and analysis of thermal and RGB data. This system setup ensures precise and reliable measurements, supporting advanced diagnostic capabilities in dynamic thermographic studies.

To achieve reliable examination results, the patient and the examination room should be prepared in accordance with the principles of thermographic examination described in the second section, based on [14]. The patient, often undergoing cardiac surgery or following a burs incident, is positioned approximately one meter from the stimulating and recording equipment. During the examination, the patient typically lies on a bed with the area of interest (e.g., the chest) exposed. Access to the patient is limited to the surface of the ROI, both for the data acquisition system and the excitation sources.

ADT requires the reconstruction of the simplest descriptive multi-exponential model parameters, mainly thermal time constants and the magnitude ΔT_s_ temperature gradients. Such descriptors are strongly correlated to thermal equivalent models, e.g., the three-layer structure described by thermal resistances R_th_(1–3) and thermal capacitances C_th_(1–3). The products R_th_(1–3)C_th_(1–3) are equivalent to thermal time constants.

**Figure 6:**
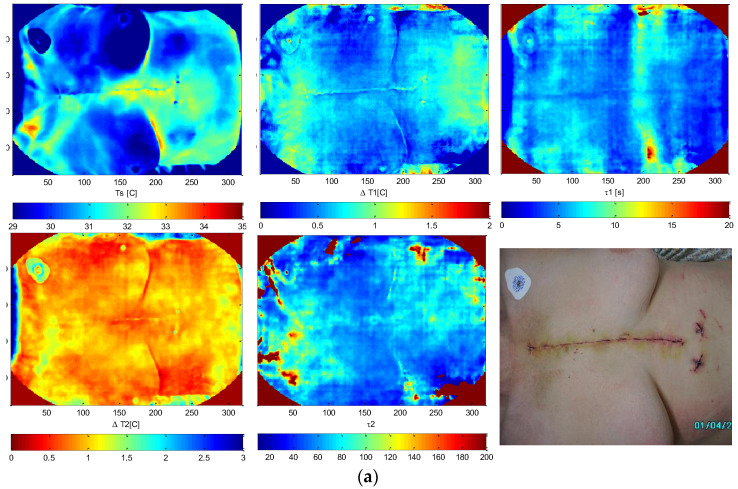

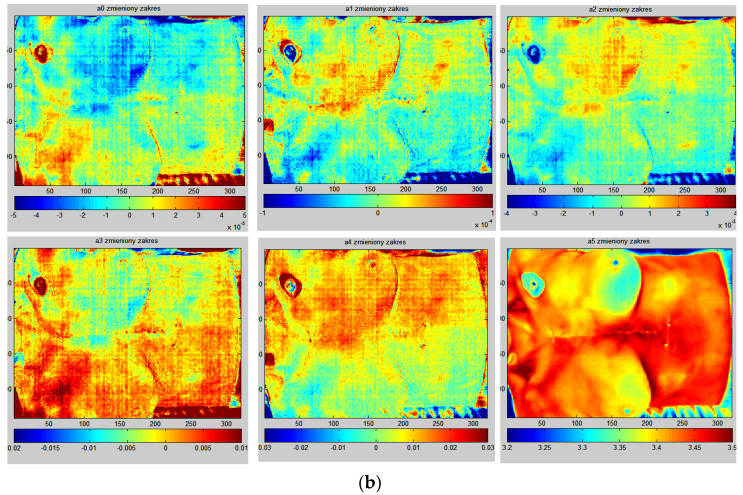
(**a**) Postoperative wound evaluation in cardiac surgery on the 3rd day after cardiac surgery; parametric images—according to Equation (2); photograph of the patient. (**b**) Postoperative wound evaluation in cardiac surgery patient on the 3rd day after cardiac surgery; TSR parametric images: first 6 parametric images of logarithmic coefficients a_i_—according to Equation (5); first row from left: a_0_, a_1_, a_2_; second row from the left: a_3_, a_4_, a_5_.

The technical solutions of such a system may vary. The use of double vision and thermographic cameras enables the acquisition of stereo vision images, e.g., used in unique solutions in medical diagnostics. The source of thermal stimulation is important. Here, two devices for cold air cryotherapy controlled by electro-valves are used. This allows for cooling the ROI more uniformly compared to a single device’s case. We had a positive experience as diagnostically valid measurements were taken after switching off the coolers, which took less than one second. Alternative solutions are possible, but the best results are for cooling. In the case of non-contact stimulation, the temperature reading is synchronized with turning the thermal source on and off, so that the tissue stimulation phase and the response phase are separate from each other. In the case of a human examination, the stimulation must be safe and must not damage tissues or cause pain. The result of the system’s operation is the acquisition of a series of thermographic images, which in the next stage are subjected to procedures specific for each method of reconstructing parameters of the thermal equivalent model of a tested object. An example is shown in Figure 6. This figure presents the results of a study described in [25], a project for optimizing cardio surgery procedures of coronary artery bypass grafting (CABG). Images of basic ADT parameters (2) (Figure 6a) and TSR parameters (5) (Figure 6b) are shown.

In conclusion, in medical diagnostics using procedures of Active Dynamic Thermography (ADT), Thermographic Signal Reconstruction (TSR), and Thermal Tomography (TT), it is recommended to use cooling as a rectangular stimulus with a duration sufficient to bring the ROI surface temperature to ambient levels. This standardizes the initial boundary conditions, ensuring a reliable interpretation of measurement results. By achieving ambient surface temperature, the return to thermodynamic equilibrium is primarily governed by internal heat flows within the ROI. This enables the accurate reconstruction of the substitute thermal model parameters, allowing for a reliable assessment of the layered structure of the tissue. In the setup shown in Figure 5, the thermographic camera can be used to automatically determine the optimal moment to stop the excitation signal (i.e., the cooling process).

Cooling to temperatures below ambient (but above 0 °C) improves measurement resolution by creating larger temperature gradients; however, the problem of the excitation pulse duration requires further studies. Keeping the ROI surface at ambient temperature for an extended time may increase the depth of tissue layers at this temperature. This may result in uncontrolled changes in the boundary conditions as well as changes in thermoregulatory processes inside the human body. To address these challenges and optimize the method, further studies are required. These should focus on refining the control of excitation pulse duration and its effects on boundary conditions to ensure more reliable and consistent diagnostic outcomes.

### 3.3. Multimodal Data Fusion

It has already been proved that fusing magnetic resonance imaging (MRI) or computed tomography (CT) with thermographic images can be useful in medical diagnostics, improving typical single-modality accuracy from 80% to even 97–98% in careful multimodality studies of breast cancer [26].

These multimodal approaches bring together the anatomical clarity of MRI/CT with the functional/metabolic cues of thermography (infrared imaging). Although the field is still emerging, a growing number of pilot studies and feasibility reports show that this combination can offer added diagnostic value, especially when we look at improved visualization approaches such as 3D Surface Alignment. MRI or CT is volumetric, so a 3D surface can be reconstructed. Thermography is usually 2D, but multi-view thermographic data can generate a 3D thermal mesh. Fusion occurs by aligning 3D meshes, enabling realistic visual overlays of temperature data on anatomical structures. These surface matching methods are used in neurosurgery, when planning surgery. The recreated 3D shape of the brain based on MRI scans is wrapped with a texture derived from thermographic imaging. We then obtain a 3D model of the temperature distribution on the brain surface, and hot spots (high temperature) may indicate a shallow arterial course or increased metabolism associated with a developing tumor [27,28].

A small number of studies have attempted to co-register the neck region in both MRI and thermographic images using surface landmarks (chin, sternal notch) or fiducial markers. Features from both modalities (e.g., nodule size from MRI, local temperature gradients from IR) and the activity of thyroid glands based on scintigraphy are then combined in a classifier to distinguish euthyroid vs. hyperthyroid states [29,30].

Modality fusion is also used in diabetic foot diagnostics for 3D reconstruction of the foot surface and in mapping facial temperature on a 3D head model from MRI/CT scans or based on surface reconstruction from a point cloud generated by depth cameras/3D scanners [31].

## 4. Machine Learning and Artificial Intelligence Methods in IR Thermal Diagnostics

**Figure 7:**
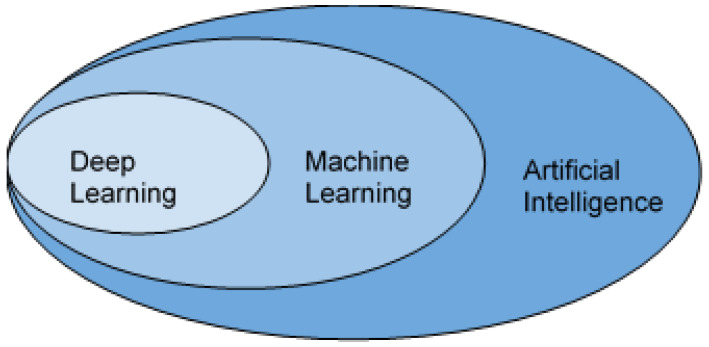
Visualization of artificial intelligence concept relationships.

The development of artificial intelligence methods, including machine learning, observed in recent years is also important in medical applications of infrared imaging. Artificial intelligence and machine learning techniques are increasingly being combined with classical methods using static and active thermography. Thermal signals have a relatively low signal-to-noise ratio, and most thermal images have a common drawback of blurred edges. Additionally, to correctly assess the properties of a thermal image, an operator with extensive knowledge related to the manual entry of various parameters is needed in order to conduct an inspection. One of the main goals of the development of artificial intelligence is to replace human work more effectively and objectively. Difficulties in analyzing thermographic images can be solved with the help of AI (artificial intelligence) technology. One of the parts of artificial intelligence techniques is deep learning, also known as hierarchical or deep machine learning. This is one of the branches of machine learning based on artificial neural networks. The learning process is called deep because the structure of artificial neural networks consists of several input, output, and hidden layers. Both machine learning (ML) and deep learning (DL) are parts of a larger field called artificial intelligence (AI) (Figure 7).

Convolutional neural networks (CNNs) are already effectively used in various fields of image processing and computer vision. They have great possibilities for feature extraction and pattern recognition in image processing. They also found applications in infrared thermal imaging in NDT—nondestructive testing. In the case of thermal images, CNNs use spatial information to detect defects, and the models that are used for this purpose include U-net, VGG, and YOLO. They provide very good results in the case of dimensionality reduction and image segmentation. In addition to detecting defects, deep learning provides the possibility of automatic estimation of defect depth. For this purpose, RNNs (recurrent neural networks), such as LSTM and GRU, are used to extract temporal features from thermal sequences, which are sensitive to the depth of defects. However, most research focuses on defect detection, segmentation, and classification [32] purposes.

Table 2 presents statistics of published scientific articles available in the IEEE Xplore database and MDPI Journals database from 2020 to 2024 regarding the application of deep learning and machine learning methods in medical diagnostics for thermographic data. The numbers below are estimates, reflecting search and filtering strategies, using keywords such as “thermography” OR “infrared imaging” AND “machine learning” OR “deep learning” OR “neural networks” AND “medical” OR “diagnostic”. Actual numbers may vary depending on search criteria, filters (e.g., conference vs. journal), and the day/time of query and research topic. e.g., is face detection on thermograms a medical application or not?

One can see a gradual increase from 2020 to 2023, reflecting the growing interest in using deep learning (DL) and machine learning (ML) for thermographic diagnostics for both databases. Looking at the IEEE Xplore results, it can be concluded that a significant number of works appear in conference proceedings (e.g., IEEE EMBC, IEEE ICIAR, etc.). Fewer but often more in-depth journal articles (e.g., *IEEE Transactions on Biomedical Engineering*, *IEEE Access*) appear each year. Breast cancer detection is a common application in thermographic ML/DL research. Other areas include vascular studies (e.g., diabetic foot), thyroid disorder detection, and sports injury monitoring. These research papers using convolutional neural networks (CNNs) remain the most commonly used architecture. Some articles explore transfer learning, while others experiment with emerging methods like Vision Transformers. While privacy concerns have prompted interest in federated learning for medical data, the actual number of articles applying FL to thermography (not only for medical applications) is relatively small but slowly increasing (1–3 articles in the 2022–2023 range).

Analyzing the data for the MDPI database, one can see a similar growing trend in publications on AI in thermal diagnostics in medicine. The most popular journals are as follows:

*Sensors*: known for publishing many articles on thermal imaging sensors, image processing, and applied machine learning; *Applied Sciences*: often features research on applied thermographic systems and image-processing algorithms; *Diagnostics*: focuses on diagnostic techniques, including AI-based medical imaging; *Cancers* (for oncology-related work), *Healthcare*, *Bioengineering*, etc.

The domain is still niche compared to broader medical imaging areas (e.g., MRI, CT), but it consistently garners interest for non-invasive, cost-effective diagnostic procedures—especially in breast cancer screening and other specialized applications. The vast majority of scientific publications on the application of artificial intelligence methods in thermographic medical diagnostics concern supporting the diagnosis of breast cancer and the diagnosis of diabetic foot in diabetics.

### 4.1. Common Data Processing in ML Approaches

**Figure 8:**
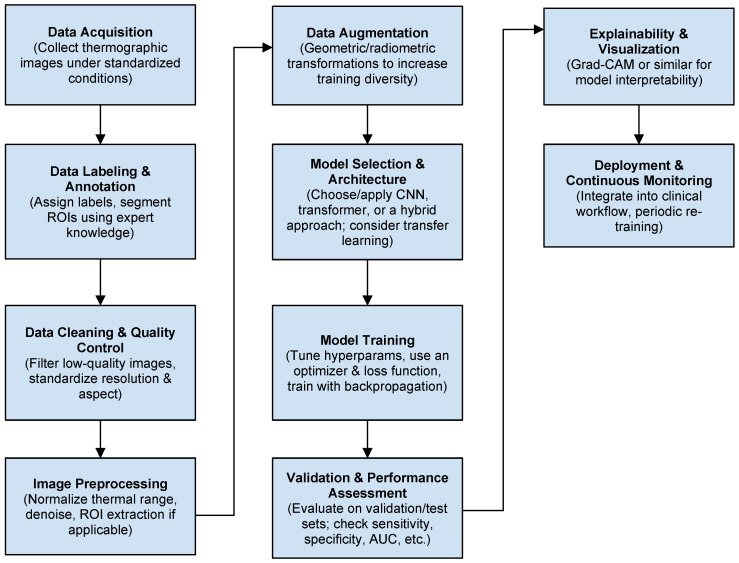
Major steps in preparing a deep learning model for thermographic diagnostics.

Below, Figure 8 is an outline of a commonly followed procedure for preparing a deep learning model to analyze thermographic (infrared) data in medical diagnostics, along with the rationale (“basis”) behind each step. While details can vary depending on the specific dataset, equipment, and clinical requirements, the general methodology remains quite consistent in medical imaging applications [33,34,35,36]. The first step is data acquisition—obtaining thermographic images from clinical settings or publicly available datasets. One should use standardized imaging protocols (e.g., controlled room temperature, patient acclimatization before imaging, fixed camera-to-subject distance, etc.). The next step is data labeling and annotation, where labels (e.g., “malignant”, “benign”, “healthy control”) are assigned to each thermogram based on the clinical diagnosis or biopsy results. Accurate labels (ideally from biopsy-confirmed cases) are essential for supervised deep learning. In some workflows, regions of interest (ROIs) (e.g., quadrants of the breast or suspicious hot spots) are segmented using expert annotations. Focusing on specific ROIs helps the model to learn localized temperature patterns correlated with malignancies. Next comes the data cleaning and quality control stage where poor-quality images (e.g., blurred, occluded, or incorrectly captured) are removed. One can also standardize resolution and aspect ratio if cameras or acquisition protocols differ across data sources.

Image processing is a crucial step for improving the whole procedure. Thermal Range Normalization means rescaling pixel intensities (temperature values) to a standard range (e.g., 0–255 if using 8-bit grayscale). Noise reduction involves applying smoothing or denoising filters (e.g., Gaussian or median filtering) to reduce sensor artifacts. It helps the network to focus on true temperature variations related to physiology. For ROI extraction, some studies use automated or semi-automated cropping around the breast area to remove the background and reduce image complexity. This improves model performance by removing irrelevant image content. Due to the small amount of data, the data augmentation procedure is necessary to train a DL model. Medical imaging datasets are often small; augmentation effectively “increases” the training set size [37]. Several operations can be used to reproduce a set of usable thermograms: Geometric Transformations: random cropping, flipping, rotation, or slight scaling of images; Radiometric Transformations: small alterations in brightness or contrast to simulate variations in sensor readings; Thermal Symmetry Approaches: e.g., for breast imaging, some studies use symmetrical flipping or merging left/right breast images to improve the model’s sensitivity to asymmetric hot spots. The central/key element of the methodology is the selection of the DL model—model selection and Architecture. Different applications require different pretrained models. One should choose a suitable architecture (e.g., CNN-based: ResNet, DenseNet, MobileNet, or transformer-based for vision tasks) and initialize with pretrained weights (from ImageNet or a large-scale thermal dataset, if available) to boost performance on limited medical data. Eventually, custom layers (e.g., fully connected layers) can be added to output binary (cancer vs. control) or multi-class predictions. During the model training stage, split the dataset into training, validation, and test sets (commonly 70%–15%–15% or similar). Then, specify hyperparameters (e.g., learning rate, batch size, number of epochs) and train using backpropagation with a suitable optimizer (e.g., Adam, SGD) and loss function (e.g., cross-entropy for classification). Monitor training with metrics like accuracy, sensitivity, specificity, and AUC (area under the ROC curve). It should be underlined that sensitivity (true positive rate) is critical in cancer detection, so focusing on these metrics ensures clinically meaningful performance.

The last three stages are related to determining the quality of the selected and trained network model. Validation, explainability, and deployment and quality monitoring and updating will ensure the effectiveness of the model’s decisions. It is necessary to evaluate the model on the validation set to tune hyperparameters (e.g., early stopping if validation loss plateaus). Test the model on an independent test set or perform cross-validation to measure generalizability with the use of clinically relevant evaluation metrics: sensitivity (recall): the probability that the model correctly identifies positive cases; specificity: the probability that the model correctly identifies negative cases; ROC/AUC and precision–recall curves: summarize performance across all possible thresholds for labeling a sample as “positive” and show model’s ability to discriminate between classes.

Model explainability is an optional but recommended step in recent experiments. Grad-CAM or similar methods are used to visualize important regions in the thermographic image for the model’s decision. Robust methodology and close collaboration with medical experts are essential to ensure accurate, explainable, and clinically viable results.

**Figure 9:**
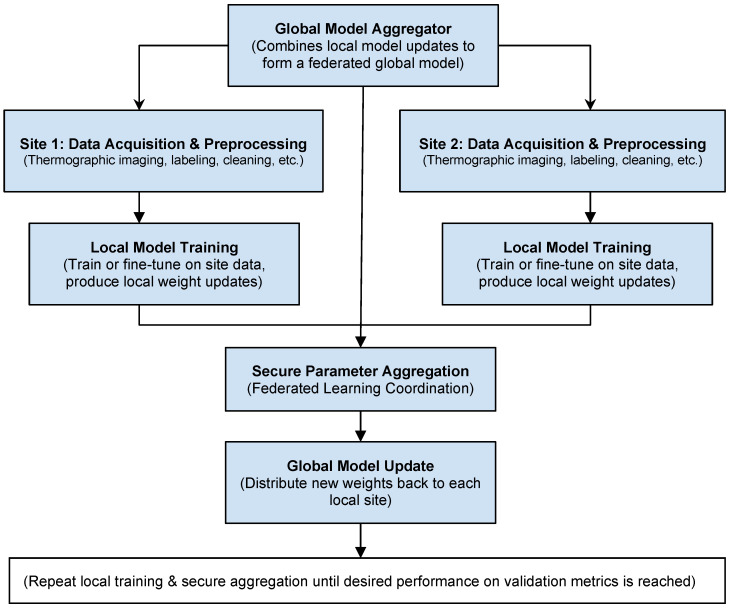
Steps in preparing a federated deep learning context for thermographic diagnostics.

In applications where data are particularly sensitive, the idea of federated learning is increasingly used. Figure 9 is a flowchart for preparing medical thermographic data for a federated learning model. In such a model, patient data never leave the hosting hospital [38,39]. The explanation of each step in a federated learning context is as follows: Each interested hospital downloads the pretrained model to their local servers. In our case, we have a global model that is downloaded by Site 1 and Site 2. This model has been pretrained on data from other federated hospitals and has predefined weights. Then, each hospital separately performs site-specific data acquisition and preprocessing—each medical facility (Site 1, Site 2, etc.) collects thermographic images under consistent protocols (room temperature, camera distance, etc.). Local teams label images (benign, malignant) and perform quality control (removing out-of-focus or incomplete images). Preprocessing steps (normalizing temperature ranges, denoising, ROI extraction) are also performed locally. In local model training, instead of sending private patient data to a central server, each site trains its own model (or fine-tunes a base model) on local data. Finally, the site then sends only the model updates (weights, gradients) to a central aggregator for secure parameter aggregation. A federated learning server (the aggregator) receives updates from multiple sites. Through secure methods (e.g., secure multi-party computation, differential privacy), the aggregator combines the local updates to create a global model without accessing raw patient data. The last step is global model updating and distribution.

After aggregation, a new global model is formed. This global model is then sent back to each site, allowing the sites to benefit from knowledge learned across all participating institutions. The process can repeat (federated learning cycles) until performance converges. Each site can also hold out local validation or test sets to check metrics like accuracy, sensitivity, specificity, and AUC. The overall performance can be assessed across sites without pooling raw patient data in one place. The model can be continuously updated in future federated learning cycles as new data are collected.

Key benefits from the federated learning model are as follows: privacy preservation—patient data never leave each institution, reducing privacy and compliance risks; collaboration without data sharing: multiple hospitals/clinics can collaboratively train a powerful model without centralizing sensitive data; scalability: additional sites can join the federated network, contributing to and benefiting from the global model; consistent updates: as each site acquires new data, future federated cycles can continually improve model performance.

### 4.2. Breast Cancer Diagnosis

Breast cancer is one of the most common and affects about 15% of women. Based on the research conducted in 2018 by the World Health Organization, over 627,000 women die from breast cancer every year [40]. Early detection of changes is crucial for effective treatment. The most common test used to make a diagnosis is mammography; unfortunately, it has side effects, such as exposing patients to radioactive X-ray radiation. The test also requires direct contact and often causes pain and discomfort to the patient. X-ray imaging is often not sufficient, and detecting pathological changes is very difficult, especially in young women, due to problems in imaging dense breast tissue. Thermography (infrared imaging) has emerged as a promising auxiliary tool for breast cancer screening due to its non-ionizing, non-invasive nature and relatively low cost compared to mammography or MRI. From 2020 to 2024, IEEE Xplore has indexed a growing body of work applying machine learning (ML) and deep learning (DL) algorithms to automate the detection of suspicious temperature anomalies associated with breast malignancies. Key motivations in these studies include improved accuracy, harnessing advanced ML and DL models for better specificity and sensitivity than traditional threshold-based methods.

Earlier works in 2020–2021 often used classical ML techniques such as Support Vector Machines (SVMs), Random Forests (RF), and k-Nearest Neighbors (k-NN) on handcrafted features (e.g., statistical measures of temperature distribution, Haralick texture features). While the performance was modest compared to newer deep models, these methods remain relevant in low-data scenarios or where computational resources are limited [41,42]. Feature engineering frequently included the following: statistical features: mean, variance, skewness of temperature distributions; texture features: GLCM-based features or wavelet-based descriptors of localized thermal patterns. The presented classic approach has its advantages, such as faster training and inference on limited hardware and the interpretability of temperature-based features. Disadvantages and limitations can also be identified: reliance on expert-driven feature extraction and potentially lower accuracy than end-to-end DL approaches.

Starting around 2021–2022, convolutional neural networks (CNNs) became the dominant approach. Researchers fine-tuned well-known architectures such as VGG, ResNet, DenseNet, and MobileNet on thermographic breast images [39,43,44,45]. Key strategies included the following: transfer learning: using ImageNet-pretrained weights to compensate for relatively small thermography datasets; data augmentation: random flips, rotations, brightness/contrast changes to improve generalization; hybrid classifiers: combining CNN feature extraction with classical ML classifiers (e.g., SVM) in a pipeline [44]. The reported benefits were as follows: higher sensitivity: some CNN-based models exceeded 90% sensitivity in detecting suspicious or malignant regions [44]; reduced false alarms: more robust feature extraction than handcrafted methods. Known challenges are as follows: limited datasets: many studies used fewer than 300–500 thermograms, risking overfitting; imbalanced classes: malignant cases are often underrepresented, requiring careful handling of class imbalance.

From 2022–2023, a few publications began exploring Vision Transformers (ViTs), originally popularized in natural image tasks [39,46]. While still in the early stages for thermography, transformers could model long-range dependencies in thermal distribution and potentially outperform CNNs on complex temperature patterns. However, the lack of large-scale thermography datasets limits the immediate success of transformer-based approaches.

Some studies extend the thermographic analysis by fusing multiple data sources (hybrid approaches and multimodal fusion), for example, mammography + thermography; MRI/CT + thermography. Hybrid models leverage complementary information—structural details from conventional imaging plus metabolic cues from thermography—to boost overall diagnostic accuracy. A popular setup involves the following: CNN or Transformer for thermography; CNN or classical ML for mammograms or ultrasound; and a late fusion layer that merges the feature vectors and performs classification. These methods often report increased sensitivity (by 2–5%) compared to single-modal setups, though complexity and data requirements are higher [47,48].

While the actual studies vary in dataset size, patient demographics, and labeling approaches (malignant vs. benign vs. healthy), the table below (Table 3) consolidates typical performance metrics reported from 2020 to 2024. The numbers are approximate and serve as a guideline to overall trends [38,49,50,51,52].

Below, one of the selected articles devoted to the diagnosis of breast cancer using DL in breast thermography is described in more detail [53]. It was found that high chemical and vascular activity of cancer tissue causes local temperature changes on the surface of the breast, which are visible on thermograms. In addition to temperature changes, physical changes in the structure of the breast, where cancer develops, such as breast asymmetry, can also be observed. The study was conducted on the Database For Mastology Research dataset, containing images taken with a thermal imaging camera from several reference points. Thermographic images obtained from 144 patients were selected for analysis. Of these, 88 were healthy, and 56 were sick or suspected of having lesions. Four pretrained convolutional neural networks, i.e., VGG16, VGG19, ResNet50, and InceptionV3, were tested on the collected images. The use of previously trained models significantly shortened the training time. Experimental studies were conducted using different test and training ratios (50% train−50% test, 70% train−30% test, 75% train−25% test). After testing, it was found that the ResNet50 network achieved the highest efficiency at the level of 88.89%. Previous studies analyzing the problem of detecting neoplastic changes in thermographic images were based on segmentation and classical machine learning methods. The model results did not prove to be better than those previously presented in the literature, but in those cases, they were based on a small number of images. Although the proposed method seems to be inefficient, it was a pioneer due to the number of images used and the methods applied [53].

Another example of using AI in breast cancer thermography examination is [54]. Statistical parameters were calculated for each breast contour and quadrant, i.e., standard deviation, median, maximum, minimum, skewness, kurtosis, entropy, area, and heat capacity. Considering that in the case of visual assessment of breast images, the symmetry of the opposite breasts is analyzed, the statistics of differences between the opposite breasts were used as input features for the neural network analysis. The data were normalized to values in the range [−1, 1]. A backpropagation (BP) neural network was used to analyze infrared breast images. Five parameters, i.e., mean, standard deviation, skewness, kurtosis, and heat capacity, whose correlation value was the highest, were extracted from the input dataset. The authors used the Levenberg–Marquardt (LM) algorithm to conduct the tests because it gave the fastest convergence and the smallest mean square error (MSE). In the article, the authors demonstrated that the use of a neural network using thermographic images is able to generate accurate output data based on the calculated statistical parameters obtained from the thermograms. The network incorrectly explained two observed cases, which could be due to the insufficient number of data available in the set, therefore it is recommended to extend the database in order to be able to expand the network and obtain the most accurate results. The authors proved that the neural network could be used to select a set of parameters that will allow for the prediction of an abnormal thermogram and the results of the neural network in combination with thermograms can complement the clinical decision and be a supporting tool for early detection of breast cancer and risk prediction. Other similar reports can be found in [55,56,57,58,59,60,61].

### 4.3. Diabetic Foot Screening

Diabetes is one of the diseases of civilization in the 21st century. It is a group of metabolic diseases characterized by hyperglycemia or increased blood sugar levels resulting from a defect in the production or action of insulin. Diabetes causes many complications and, if improperly treated, can lead to the failure of many systems and organs. An example is the so-called diabetic foot, which is a set of specific ailments affecting the feet of patients with long-term, poorly treated diabetes. The disease leads to disorders in the blood supply and innervation of the foot, which can ultimately lead to ulceration, tissue necrosis, and, in the most critical cases, amputation of the foot. Changes occurring in the foot are characterized by changes in the temperature distribution in the plantar part. Such abnormal changes can be observed on thermographic images, and their analysis allows for the rapid identification of patients at risk of diabetic foot syndrome at an early stage of its development. Diabetic foot ulcer (DFU) is one of the most serious complications of diabetes, often leading to infection, hospitalization, and even amputation. Thermography can capture temperature anomalies (hot or cold spots) that can precede visible ulceration. Deep learning (DL) models can be trained on foot thermographic images to identify early-stage DFU risk by detecting subtle temperature asymmetries. In [62], deep learning methods were proposed to analyze foot thermograms for detecting diabetic feet. Six deep CNN models were tested on thermograms containing an image of a single foot, but DenseNet201 outperformed other networks with an overall sensitivity of about 94%. In the next step, it was tested whether using thermograms with images of both feet improves the detection of diabetic foot. It was found that such thermograms with the use of Gamma enhancement outperform other methods, and the best among the tested networks was the shallow MobilenetV2 network. The higher performance obtained using a combination of images can be explained by the fact that such thermograms contain more distinguishable features. The performance of 10 classical classifiers was also tested with three feature selection techniques and different combinations of feature optimization. All possible combinations of classifiers and feature selection techniques were tested. The analysis showed that the best results were given by the Adaboost classifier with a Random Forest function. The results were even higher than the tested deep learning techniques. This network can be easily implemented in a smartphone application, which will allow the user to monitor their condition even at home. This provides great opportunities for the quick and non-invasive diagnosis of complications caused by diabetes [62]. Other similar investigations are presented, e.g., in [63,64,65,66,67,68,69,70,71,72,73,74]. The most popular CNN models used in diabetic foot diagnosis are DenseNet, CNN-based transfer learning techniques, Deep Residual Networks, Mobile-Net, and Vision Transformers. Federated learning was also implemented for this purpose [74].

### 4.4. Other Medical Applications of Thermography and DL/ML

From 2020 to 2024, IEEE Xplore saw a surge of ML/DL research leveraging thermographic imaging to diagnose musculoskeletal injuries, vascular disorders, rheumatologic conditions, and thyroid dysfunction and for fever screening (including COVID-19). The work concerns, for example, monitoring driver fatigue, breathing problems in newborns, and discopathy. Deep CNNs (ResNet, DenseNet, VGG) dominate as the go-to architectures, often with transfer learning to handle small datasets.

Despite promising results—commonly achieving 80–90% accuracy in pilot studies—challenges related to dataset sizes, standardization, and clinical validation remain. Future work aims to expand open datasets, integrate multimodal data, and explore federated learning to scale thermographic diagnostics beyond localized pilot studies [75,76,77,78,79,80,81,82,83,84,85,86,87].

The whole world struggled with the COVID-19 pandemic in 2019–2023. In order to prevent the rapid spread of diseases, prevention systems were introduced, consisting of monitoring the temperature of people moving to public spaces such as airports, public buildings, hospitals, etc. Thermographic systems were used, supported by DL/ML analyses of facial thermograms. Algorithms were used in the analysis of thermograms to segment the facial area on thermograms and characteristic facial structures such as the forehead, eyes, mouth, and cheeks. The average temperature was determined in these regions to detect people with a fever. Examples of articles devoted to COVID-19 screening are [85,86,87,88,89,90]. These articles collectively illustrate how ML and DL techniques—ranging from classical machine learning with feature extraction to transfer learning and multimodal CNNs—have been adapted for COVID-19 fever screening and temperature-based monitoring. While thermography cannot independently diagnose COVID-19, it remains a practical, non-contact method for identifying potential feverish individuals for further testing. In [86], the authors propose a CNN-based pipeline for locating the inner canthus of the eye (one of the most stable references for core temperature in thermographic images). They then apply threshold-based analysis to classify individuals as “fever” vs. “no fever” and achieved over 90% sensitivity and ~85% specificity when testing in a large indoor setting, noting that ambient temperature and physical exertion can lead to false positives. In [85], the system captures frontal thermal images, extracts temperature features from facial regions, and feeds them to an SVM for classification. The authors also explore data augmentation techniques to handle small training sets. They demonstrated 85–90% accuracy in detecting febrile vs. non-febrile subjects. An interesting approach presented in [89] is the proposal to use a multimodal IR + RGB system that combines infrared (IR) and visible-light (RGB) camera feeds in a multimodal framework to better detect feverish states in large crowds. A two-stream CNN architecture extracts features from both thermal and RGB images; a late fusion layer merges the feature vectors for the final classification of “possible fever”. The authors report higher accuracy (~93%) and precision than a thermal-only system, credited to the context provided by RGB (e.g., confirming if face masks or hair might be interfering with the thermal readings). Daily/weekly thermal changes in symptomatic COVID-19 patients were investigated to see if IR imaging can detect the progression or early improvement of fever-related patterns, as reported in [90]. A recurrent neural network (LSTM) takes sequences of facial IR frames over multiple days, aiming to classify changes in temperature distribution over time. There was some correlation between persistently elevated facial temperature and a more severe clinical course. The model flagged potential deterioration earlier than standard single-timepoint checks. Such a system could be integrated into a telehealth system for quarantined or hospitalized patients needing regular fever check-ins. There are also works that address the issue of eliminating artifacts such as masks, which make it difficult to determine the facial area in thermograms [91,92,93].

### 4.5. Image Processing for Medical Applications of Thermography and DL/ML

Deep learning (DL) and machine learning (ML) methods have been applied to improve the quality of thermographic images. These examples range from noise reduction and super-resolution to artifact correction and contrast enhancement.

- Denoising with Autoencoders [94,95]

Autoencoders can learn a latent representation of “clean” thermal images and reconstruct denoised outputs by minimizing the difference between noisy input and the ground-truth (clean) image. In practice, you collect pairs of noisy and (ideally) noise-free thermograms or synthetically add noise to relatively clean images for training.

- Super-Resolution via Generative Adversarial Networks (GANs) [96]

GAN-based super-resolution aims to reconstruct high-resolution (HR) thermographic images from low-resolution (LR) inputs. In a pix2pix or SRGAN framework, a Generator tries to upscale LR thermograms, while a Discriminator forces the Generator to produce more realistic, detail-rich images. The main key benefit is enhanced detail visibility—better detection of subtle thermal anomalies or vascular patterns—and the second one is enabling lower-cost IR cameras to produce effectively higher-resolution images for diagnostic purposes.

- Artifact Removal with Deep CNNs [97]

Thermographic images often contain artifacts (e.g., reflections, occlusions or lens smudges) that degrade image quality. Convolutional neural networks—especially encoder–decoder structures—can learn to identify and in paint or remove these artifacts. Artifacts can mask or mimic hot/cold spots, leading to misdiagnosis.

- Contrast Enhancement using DL-Based Image-to-Image Translation [98]

Low contrast in thermography can obscure small temperature differences. Image-to-Image translation networks (e.g., pix2pix, CycleGAN) can be adapted to improve contrast and highlight subtle variations in temperature—warmer or cooler areas become more distinguishable.

- Multi-Frame Fusion/Spatio-Temporal Enhancement [99,100]

In sequences of thermographic images, ML/DL methods combine multiple frames to create a single higher-quality image or a more stable reading. This is particularly relevant when patients move slightly during imaging or sensor noise changes over time.

Deep learning and machine learning methods have demonstrated substantial capabilities in thermographic image quality improvement through denoising, super-resolution, artifact removal, contrast enhancement, and spatio-temporal fusion. These enhancements are crucial for medical diagnostics, as clearer and more detailed thermograms help clinicians identify subtle signs of disease more confidently.

### 4.6. Medical Databases of Thermograms

Thermographic data for medical ML/DL are still fragmented. The best approach is to combine multiple small databases or engage in collaborative or federated efforts to gather enough diversity and volume for robust model training and evaluation. In comparison to fields like medical ultrasound or mammography, thermography generally lacks massive, open-access repositories of thousands or tens of thousands of annotated images. The “largest” in thermography might only have 200–600 images that are richly annotated with clinical metadata (biopsy results, ROI segmentations, patient demographics). Annotation quality can vary significantly: some sets provide pixel-level masks, while others only give patient-level labels (e.g., “malignant”, “benign”, or “healthy”). Most of these resources require direct communication with the dataset owners (e.g., emailing the authors) or filling out data-use agreements to respect patient privacy and ethics. Below are the most popular thermogram databases:

1. DMR-IR (Dynamic Infrared) Breast Thermography Database [101]—official “one-click” URL: http://visual.ic.uff.br/dmi (accessed on 25 January 2025). It is commonly obtained by emailing the original dataset authors or lab. It includes around 200–250 breast thermographic images from approximately 100 subjects (numbers can vary depending on the dataset version). It includes a mix of benign, malignant, and healthy cases. Annotations are as follows: typically accompanied by ROI markings indicating suspicious regions; metadata may include patient history, biopsy results, or clinical findings (depending on the version).
2. BIR/DMR Database (Breast Thermography) [102]—ranges from 92 to 150+ thermographic images, focusing on breast cancer screening. Annotations are as follows: may include segmentation masks or bounding boxes around suspicious hot spots or recognized lesions, often validated by radiologists or breast specialists.
3. STANDUP Thermographic Foot Ulcer Database [103]—a research database consisting of 415 multispectral images (thermal and RGB images) of the plantar foot from healthy (125 images) and diabetic subjects (290 images). The healthy subjects were members of two research laboratories (PRISME in France and IRF-SIC in Morocco). The second group was composed of type II diabetic patients who participated in an acquisition campaign at the Hospital Nacional Dos de Mayo in Lima, Peru, as part of a study on the early detection of ulcers in patients with diabetic foot.
4. Breast Thermography Database, San Juan de Dios Hospital, Consultorio Rosado (Cali, Colombia) [104]. This dataset was acquired inside a medical office with dimensions at a temperature of 22–24 °C and relative humidity of 45–50 %. A FLIR A300 camera was used to capture the images. The American Academy of Thermology (AAT) protocol was used to prepare the patient and image capture. Images captured were from 119 patients. A set of three images was taken in the chest area of each patient. The ages of the patients ranged from 18 to 81 years. The pathologies related by each patient were benign (BP) and malignant (MP); otherwise, the breast status was normal (N). Each set of images (anterior, left, and right oblique positions) has the associated diagnosis of each breast and the weight, height, temperature, and age of the patient. The medical diagnosis was obtained from the pathology report obtained a few days after the thermography was performed.

Because such ML/DL techniques are still relatively niche, much of the work appears as pilot studies or proof-of-concept papers rather than large-scale clinical trials. It is therefore difficult to refer to the clinical effectiveness of the described methods and solutions. Nevertheless, some reports try to summarize the benefits of using these innovative and developmental algorithms. In [105,106], an attempt was made to estimate the benefits of using ML/DL in thermographic screening of women with suspected breast cancer. It was shown [105] that for the used thermal imaging and classification system, the test device had a sensitivity of 82.5% (95% CI 73.2 to 91.9) and specificity of 80.5% (95% CI 75.0 to 86.1) as compared with a diagnostic mammogram, which had a sensitivity of 92% (95% CI 80.7 to 97.8) and specificity of 45.9% (95% CI 34.3 to 57.9) when BI-RADS 3 (Breast Imaging-Reporting and Data System) was considered as test-positive. The overall area under the curve (AUC) was 0.845. For women aged <45 years, the test device had a sensitivity and specificity of 87.0% (95% CI 66.4 to 97.2) and 80.6% (95% CI 72.9 to 86.9), respectively. For women aged ≥45 years, the sensitivity and specificity were 80.5% (95% CI 65.1 to 91.2) and 86.5% (95% CI 78.0 to 92.6, respectively). Moreover, the final conclusion [105] is that the thermographic diagnostic system showed acceptable sensitivity and specificity with respect to mammography in the overall patient population. It even outperformed mammography in women with dense breasts and those reported as BI-RADS 0.

## 5. Discussion and Practical Advice

Machine learning and deep learning have reinvigorated thermography, once viewed skeptically as an adjunct to mainstream imaging. Today, these methods provide a comprehensive feature extraction and robust classification pipeline that can detect thermal anomalies indicative of cancer, infection, inflammation, and other pathologies. Based on the literature studies, we do observe continuous progress in ML and DL for medical thermography, especially in the following:

(1) Improved Accuracy and Sensitivity: Deep CNN architectures (e.g., ResNet, DenseNet) have significantly enhanced classification and lesion detection accuracy in small yet specialized thermographic datasets (breast, diabetic foot, musculoskeletal conditions, etc.). Transfer learning (pretraining on large image datasets like ImageNet) is widely used to overcome the data scarcity typical in thermographic imaging tasks. Explainability (e.g., via Grad-CAM) is increasingly important in medical contexts, building trust among clinicians.
(2) Enhanced Image Quality and Feature Extraction: Autoencoders, GAN-based super-resolution, and denoising networks are improving thermogram clarity, making subtle temperature contrasts more detectable; ROI-based and segmentation-focused methods (e.g., U-Net variants) help zoom in on clinically significant areas, improving local lesion detection.
(3) Growing Body of Proof-of-Concept Studies: Much of the literature consists of pilot or feasibility studies with relatively small datasets. Despite promising initial results, multicenter trials and standardized protocols remain limited.
(4) Adoption Across Multiple Pathologies: While breast cancer and diabetic foot ulcers have been the most studied, recent work extends to arthritis, thyroid disorders, fever screening, and sports/musculoskeletal injuries—highlighting thermography’s versatility when combined with AI.

Of course, there are also disadvantages and difficulties in implementing ML and DL methods in medical applications. This applies primarily to areas such as the following:

(1) Data Scarcity and Heterogeneity: Most thermography studies rely on small, proprietary datasets with inconsistent acquisition protocols (room temperature, camera brand, etc.). The lack of standardized open-source datasets limits reproducibility and large-scale validation.
(2) Variability in Imaging Protocols: Even slight changes in patient positioning, camera angle, or ambient temperature can affect thermographic readings, impacting model generalizability.
(3) Regulatory and Clinical Acceptance: For thermography-based AI tools to become standard in clinical practice, they need extensive validation and compliance with medical device regulations.
(4) Complexity of Models: Large models (ResNet50 and beyond) risk overfitting to limited thermal data, requiring extensive augmentation or additional data.

It is not easy to predict the future, but it can be assumed that the development will go in the following directions, as is observed for other AI applications in image processing and process monitoring:

(1) Federated Learning and Collaborative Data Sharing: To address small, fragmented datasets, federated learning will enable multiple clinics to train shared models without pooling sensitive data. This approach can increase dataset diversity, improving model robustness and generalization.
(2) Multimodal Fusion: Combining thermography with conventional imaging (e.g., mammography, ultrasound) or even electronic health records could yield superior diagnostic confidence. Late-fusion or attention-based networks will combine different data sources while highlighting clinical insights.
(3) Explainable AI (XAI) and Regulatory Push: As AI-based diagnostics near clinical deployment, interpretability will be paramount for regulatory approval and clinician trust.
(4) Standardized Protocols and Larger Databases: We can expect a push toward standardizing thermographic acquisition (e.g., patient preparation, camera calibration) across multiple institutions. Also, the data format should be standardized, e.g., DICOM for thermography examination as a mandatory standard. Larger, well-annotated datasets—potentially released by research consortia—will pave the way for more robust AI models.
(5) Real-time Monitoring and Telemedicine: Wearable or continuous thermographic sensors in hospitals or home environments could monitor temperature changes over time, detecting early warning signs of infection or inflammation. Telehealth may integrate these data streams, allowing remote specialists or AI systems to flag emerging issues.

While dataset size, protocol standardization, and clinical validation remain bottlenecks, the field is rapidly evolving. Over the next 5–10 years, we can expect the following:

- Progress in accuracy, resolution, and sensitivity of new equipment at lower prices.
- Scalable, privacy-preserving collaborations via federated learning.
- Real-time, embedded AI solutions on portable or wearable devices.
- Greater acceptance from clinicians and regulatory bodies as interpretability tools mature.
- The new medical-application-oriented market offering handy and versatile systems with software allowing the integration of multimodality images, including multispectral sensors and advanced data treatment.

## Figures and Tables

**Table 1:** Approximate year of technology progress in IR thermal imaging in medicine.

Years	Technology Progress
1958–1960	single-detector scanning camera, 1 image/minute, analog signal
~1965	InSb cooled detector, MWIR, scanning mirrors, 30 images/s
~1970	HgCdTe cooled detector, LWIR, scanning
~1975	digital signal, isotherms, 60 images/s, real-time images
~1985	FPA detectors and integrated software in hand-held cameras
~1990	uncooled FPA arrays
Now	introduction of high-resolution, uncooled thermal imagers, multimodality systems, multispectral and high-resolution arrays, advanced AI tools on board IR thermal systems

**Table 2:** Approximate publication counts (2020–2024) for IEEE Xplore and MDPI databases.

Year	IEEE Xplore	MDPI Journals
2020	10–15	5–10
2021	20–25	10–15
2022	30–35	15–25
2023	40–50	25–35
2024	15–25	15–25 ^1^

^1^ The data for 2024 may be incomplete (in-press or early-access articles might not yet be fully indexed or discoverable).

**Table 3:** Key performance metrics (accuracy, sensitivity, specificity, AUC) and common trends found in studies published between 2020 and 2024 for breast cancer screening.

Model	Dataset Size	Accuracy	Sensitivity	Specificity	AUC	Notes
VGG16	~300–600 images	78–85%	80–88%	75–83%	0.80–0.85	Often used as a baseline, overfitting risks, especially in small data.
ResNet50	~500–1000 images	85–90%	88–92%	82–88%	0.86–0.92	Residual blocks enable deeper feature extraction, typically strong performance.
DenseNet121	~500–1000 images	85–91%	88–94%	80–90%	0.86–0.93	Dense connections can boost feature reuse, good for relatively small data.
MobileNet	~300–500 images	78–85%	78–88%	75–83%	0.78–0.85	Lightweight model appealing for bedside or low-power devices.
ViT	~300–700 images	80–88%	83–90%	78–85%	0.84–0.89	Emerging approach; efficacy limited by dataset size (transfer learning is key).

Note: Exact figures differ across papers; table values are approximate averages from multiple small-scale studies.
